# Supplementary material for: The drivers and functions of rock juggling in otters
Source: R Soc Open Sci. 2020 May 6;7(5):200141. doi: 10.1098/rsos.200141 (PMC7277247; doi:10.1098/rsos.200141)
Supplement: Supplementary tables S1-6 [file rsos200141supp1.docx]

Table S1. Group composition of otters at New Forest Wildlife Park, Newquay Zoo and Tamar Otter and Wildlife Centre.

| **Group ID** | **Site** | **Species** | **Sex** | **Age (years)** |
| --- | --- | --- | --- | --- |
| NF1 | New Forest Wildlife Park | ASC | M | 19 |
|  |  |  | M | 13 |
|  |  |  | F | 18 |
|  |  |  | F | 14 |
| NF2 | New Forest Wildlife Park | ASC | M | 14 |
|  |  |  | M | 12 |
|  |  |  | M | 12 |
|  |  |  | F | 13 |
| NF3 | New Forest Wildlife Park | ASC | M | 6 |
|  |  |  | F | 14 |
| NF4 | New Forest Wildlife Park | ASC | M | 15 |
| NQ1 | Newquay Zoo | ASC | M | 9 |
|  |  |  | M | 1 |
|  |  |  | M | 8 months |
|  |  |  | F | 8 |
|  |  |  | F | 6 |
|  |  |  | F | 2 |
|  |  |  | F | 1 |
|  |  |  | F | 1 |
|  |  |  | F | 1 |
|  |  |  | F | 8 months |
|  |  |  | F | 8 months |
|  |  |  | F | 8 months |
| T1 | Tamar Otter and Wildlife Centre | ASC | M | 9 |
|  |  |  | M | 6 |
|  |  |  | M | 4 |
|  |  |  | M | 2 |
|  |  |  | M | 2 |
|  |  |  | M | 1 |
|  |  |  | M | 6 months |
|  |  |  | F | 11 |
|  |  |  | F | 6 |
|  |  |  | F | 4 |
|  |  |  | F | 2 |
|  |  |  | F | 2 |
|  |  |  | F | 1 |
|  |  |  | F | 6 months |
|  |  |  | F | 6 months |
| T2 | Tamar Otter and Wildlife Centre | ASC | M | 6 |
|  |  |  | M | 5 |
|  |  |  | M | 5 |
| T3 | Tamar Otter and Wildlife Centre | ASC | M | 7 |
|  |  |  | F | 8 |
|  |  |  | F | 3 |
| NF5 | New Forest Wildlife Park | SCO | M | 5 |
|  |  |  | M | 3 months |
|  |  |  | F | 4 |
|  |  |  | F | 3 months |
| NF6 | New Forest Wildlife Park | SCO | F | 4 |
|  |  |  | F | 1 |

Table S2. Ethogram of behaviours recorded

| **Behaviour** | **Description** |
| --- | --- |
| Walk | Slow, 4 beat gait |
| Run | Fast, lolloping gait |
| Lie | Belly fully in contact with ground, head raised and alert |
| Rest | Laying with head on ground, eyes may be open or closed |
| Sit up | Sat on rump, body curled up (stomach contracted), hind feet elevated off ground, fore paws grasping hind feet |
| Stand | Standing with all 4 feet in contact with the ground or similar surface |
| Sentinel | Standing upright on hind legs |
| Autogroom | Cleaning or scratching self with paws and/or mouth |
| Allogroom | Cleaning conspecific with mouth/paws |
| Beg | At fence, face and/or paws through the fence, intense vocalisations, may be standing up or lying |
| Rub | Lying on ground, moving side to side, scrubbing side, back and/or front of body on ground |
| Scrabble | Padding ground with front paws, head up |
| Feed | Manipulating and consuming food items |
| Chew | Applying and releasing pressure with jaws on typically inedible item. |
| Forage | Scrabbling through substrate and snuffling ground with nose |
| Drink | At edge of or in water, mouth in water, swallowing, making ripples |
| Nursing | Mother feeding cubs, encouraging them to suckle |
| Suckling | Cubs feeding from teat of mother |
| Swim | Body submerged, moving through water, head may be submerged or above surface |
| Playfight | Grasping/rolling around with conspecific in water or on land |
| Rock juggle | Manipulating pebble(s) in paws and mouth, possibly throwing pebble in air and catching |
| RJ on back | Rock juggling on back, throwing and catching pebbles, rolling pebbles across chest |
| RJ face forward | Rock juggling while laying, passing pebble between paws across ground |
| RJ roll | Rock juggling while rolling, typically only happens in water |
| RJ against wall | Rock juggling against a wall (or similar surface) throwing and catching pebbles off of surface and pushing pebbles up surface |
| RJ sentinel | Typically seen in SCO. Standing upright and throwing item into the air, attempts made to catch item. |
| Dig | Displacing substrate with paws, creating a hole |
| Scent mark | Arched back, posterior raised above ground, 2-step "dance" with back feet, tail sweep |
| Mating | Male mounting a female and thrusting rapidly |
| Mount | Facing same direction as conspecific with torso on top of conspecific, hind quarters tucked close to hind quarters of conspecific. Fore arms used to grip waist of conspecific. Behaviour can be seen in males and females. |

Table S3. A rotational schedule that was used for observations conducted at new forest wildlife park. Each rotation was repeated three times to yield 12 hours of observations.

| **Time** | **Scheduled Feed Times** | **Group Rotations** | | | | | |
| --- | --- | --- | --- | --- | --- | --- | --- |
| 1000 – 1100 | None | Group 1 | Group 2 | Group 3 | Group 4 | Group 5 | Group 6 |
| 1100 – 1200 | 1130  (Group 1 and 2) | Group 2 | Group 1 | Group 4 | Group 3 | Group 6 | Group 5 |
| 1200 – 1300 | 1230  (Group 3 – 6) | Group 3 | Group 4 | Group 1 | Group 2 | Group 5 | Group 6 |
| 1300 - 1400 | None | Group 4 | Group 3 | Group 2 | Group 1 | Group 6 | Group 5 |

Table S4. Post hoc results for latency to first interact with puzzles depending on species, the type of puzzle and the order in which puzzles were presented. NAs represent missing data.

| **Species (ASC vs. SCO)** | | | | | | |
| --- | --- | --- | --- | --- | --- | --- |
| **Puzzle type** | | **Puzzle order** | **Diff** | **Lwr** | **Upr** | **P adj** |
| Tennis balls | | First | NA | NA | NA | NA |
|  |  | Second | NA | NA | NA | NA |
|  |  | Third | 12.467 | -8.388 | 33.321 | 0.782 |
| Bottles | | First | 0.500 | -23.492 | 24.492 | 1.000 |
|  |  | Second | -0.684 | -17.896 | 16.527 | 1.000 |
|  |  | Third | NA | NA | NA | NA |
| Bricks | | First | NA | NA | NA | NA |
|  |  | Second | -1.421e-14 | -33.930 | 33.930 | 1.000 |
|  |  | Third | 1.286 | -16.079 | 18.650 | 1.000 |
| **Puzzle types presented first** | | | | | | |
| ASC | Tennis balls vs. Bottles | | NA | NA | NA | NA |
|  | Tennis balls vs. Bricks | | NA | NA | NA | NA |
|  | Bottles vs. Bricks | | 2.289 | -12.951 | 17.530 | 1.000 |
| SCO | Tennis balls vs. Bottles | | -42.000 | -65.992 | -18.008 | 0.016e-4 |
|  | Tennis balls vs. Bricks | | NA | NA | NA | NA |
|  | Bottles vs. Bricks | | NA | NA | NA | NA |
| **Puzzle types presented second** | | | | | | |
| ASC | Tennis balls vs. Bottles | | -0.316 | -11.139 | 10.508 | 1.000 |
|  | Tennis balls vs. Bricks | | -3.000 | -32.056 | 26.056 | 1.000 |
|  | Bottles vs. Bricks | | -2.684 | -31.108 | 25.739 | 1.000 |
| SCO | Tennis balls vs. Bottles | | NA | NA | NA | NA |
|  | Tennis balls vs. Bricks | | NA | NA | NA | NA |
|  | Bottles vs. Bricks | | -2.000 | -27.290 | 23.290 | 1.000 |
| **Puzzle types presented third** | | | | | | |
| ASC | Tennis balls vs. Bottles | | 1.236 | -9.262 | 11.734 | 1.000 |
|  | Tennis balls vs. Bricks | | -0.819 | -13.500 | 11.862 | 1.000 |
|  | Bottles vs. Bricks | | -2.055 | -15.043 | 10.933 | 1.000 |
| SCO | Tennis balls vs. Bottles | | NA | NA | NA | NA |
|  | Tennis balls vs. Bricks | | -12.000 | -35.992 | 11.992 | 0.939 |
|  | Bottles vs. Bricks | | NA | NA | NA | NA |
| **Puzzle order for tennis balls** | | | | | | |
| ASC | First vs. Second | | NA | NA | NA | NA |
|  | First vs. Third | | NA | NA | NA | NA |
|  | Second vs. Third | | -1.467 | -12.777 | 9.843 | 1.000 |
| SCO | First vs. Second | | NA | NA | NA | NA |
|  | First vs. Third | | -29.000 | -52.992 | -5.008 | 0.004 |
|  | Second vs. Third | | NA | NA | NA | NA |
| **Puzzle order for bottles** | | | | | | |
| ASC | First vs. Second | | 2.184 | -13.056 | 17.425 | 1.000 |
|  | First vs. Third | | 2.269 | -13.571 | 18.110 | 1.000 |
|  | Second vs. Third | | 0.085 | -9.887 | 10.057 | 1.000 |
| SCO | First vs. Second | | 1.000 | -24.290 | 26.290 | 1.000 |
|  | First vs. Third | | NA | NA | NA | NA |
|  | Second vs. Third | | NA | NA | NA | NA |
| **Puzzle order for bricks** | | | | | | |
|  | | | **Diff** | **Lwr** | **Upr** | **P adj** |
| ASC | First vs. Second | | -2.789 | -31.213 | 25.634 | 1.000 |
|  | First vs. Third | | -2.075 | -14.324 | 10.174 | 0.999 |
|  | Second vs. Third | | 0.714 | -28.902 | 30.331 | 1.000 |
| SCO | First vs. Second | | NA | NA | NA | NA |
|  | First vs. Third | | NA | NA | NA | NA |
|  | Second vs. Third | | 2.000 | -21.992 | 25.992 | 1.000 |

Table S5. Post hoc results for time spent interacting with puzzles depending on species, the type of puzzle and the order in which puzzles were presented. NAs represent missing data.

| **Species (ASC vs. SCO)** | | | | | | |
| --- | --- | --- | --- | --- | --- | --- |
| **Puzzle type** | | **Puzzle order** | **Diff** | **Lwr** | **Upr** | **P adj** |
| Tennis balls | | First | NA | NA | NA | NA |
|  |  | Second | NA | NA | NA | NA |
|  |  | Third | 83.567 | -426.242 | 593.375 | 1.000 |
| Bottles | | First | 39.750 | -546.758 | 626.258 | 1.000 |
|  |  | Second | 8.018 | -412.726 | 428.761 | 1.000 |
|  |  | Third | NA | NA | NA | NA |
| Bricks | | First | NA | NA | NA | NA |
|  |  | Second | -8.500 | -837.948 | 820.948 | 1.000 |
|  |  | Third | -81.429 | -505.912 | 343.055 | 1.000 |
| **Puzzle types presented first** | | | | | | |
| ASC | Tennis balls vs. Bottles | | NA | NA | NA | NA |
|  | Tennis balls vs. Bricks | | NA | NA | NA | NA |
|  | Bottles vs. Bricks | | 244.855 | -127.708 | 617.419 | 0.638 |
| SCO | Tennis balls vs. Bottles | | -515.250 | -1101.758 | 71.258 | 0.157 |
|  | Tennis balls vs. Bricks | | NA | NA | NA | NA |
|  | Bottles vs. Bricks | | NA | NA | NA | NA |
| **Puzzle types presented second** | | | | | | |
| ASC | Tennis balls vs. Bottles | | -9.684 | -274.269 | 254.901 | 1.000 |
|  | Tennis balls vs. Bricks | | 7.000 | -703.297 | 717.297 | 1.000 |
|  | Bottles vs. Bricks | | 16.684 | -678.151 | 711.519 | 1.000 |
| SCO | Tennis balls vs. Bottles | | NA | NA | NA | NA |
|  | Tennis balls vs. Bricks | | NA | NA | NA | NA |
|  | Bottles vs. Bricks | | 0.167 | -618.067 | 618.400 | 1.000 |
| **Puzzle types presented third** | | | | | | |
| ASC | Tennis balls vs. Bottles | | 56.297 | -200.331 | 312.926 | 1.000 |
|  | Tennis balls vs. Bricks | | 221.495 | -88.504 | 531.494 | 0.489 |
|  | Bottles vs. Bricks | | 165.198 | -125.298 | 482.693 | 0.916 |
| SCO | Tennis balls vs. Bottles | | NA | NA | NA | NA |
|  | Tennis balls vs. Bricks | | 56.500 | -530.008 | 643.008 | 1.000 |
|  | Bottles vs. Bricks | | NA | NA | NA | NA |
| **Puzzle order for tennis balls** | | | | | | |
| ASC | First vs. Second | | NA | NA | NA | NA |
|  | First vs. Third | | NA | NA | NA | NA |
|  | Second vs. Third | | -37.067 | -313.549 | 239.416 | 1.000 |
| SCO | First vs. Second | | NA | NA | NA | NA |
|  | First vs. Third | | -505.750 | -1092.258 | 80.758 | 0.180 |
|  | Second vs. Third | | NA | NA | NA | NA |
| **Puzzle order for bottles** | | | | | | |
| ASC | First vs. Second | | -6.934 | -379.498 | 365.629 | 1.000 |
|  | First vs. Third | | 21.981 | -365.247 | 409.208 | 1.000 |
|  | Second vs. Third | | 28.915 | -214.849 | 272.679 | 1.000 |
| SCO | First vs. Second | | -38.667 | -656.900 | 579.567 | 1.000 |
|  | First vs. Third | | NA | NA | NA | NA |
|  | Second vs. Third | | NA | NA | NA | NA |
| **Puzzle order for bricks** | | | | | | |
|  | | | **Diff** | **Lwr** | **Upr** | **P adj** |
| ASC | First vs. Second | | -235.105 | -929.940 | 459.730 | 0.999 |
|  | First vs. Third | | -57.677 | -357.113 | 241.759 | 1.000 |
|  | Second vs. Third | | 177.429 | -546.573 | 901.430 | 1.000 |
| SCO | First vs. Second | | NA | NA | NA | NA |
|  | First vs. Third | | NA | NA | NA | NA |
|  | Second vs. Third | | 104.500 | -482.008 | 691.008 | 1.000 |

Table S6. Post hoc results for time spent interacting with puzzles before successfully solve them depending on species, the type of puzzle and the order in which puzzles were presented. NAs represent missing data.

| **Species (ASC vs. SCO)** | | | | | | |
| --- | --- | --- | --- | --- | --- | --- |
| **Puzzle type** | | **Puzzle order** | **Diff** | **Lwr** | **Upr** | **P adj** |
| Tennis balls | | First | NA | NA | NA | NA |
|  |  | Second | NA | NA | NA | NA |
|  |  | Third | 162.231 | -81.653 | 406.114 | 0.582 |
| Bottles | | First | 11.750 | -251.002 | 274.502 | 1.000 |
|  |  | Second | 6.577 | -171.928 | 185.082 | 1.000 |
|  |  | Third | NA | NA | NA | NA |
| Bricks | | First | NA | NA | NA | NA |
|  |  | Second | -63.000 | -395.357 | 269.357 | 1.000 |
|  |  | Third | NA | NA | NA | NA |
| **Puzzle types presented first** | | | | | | |
| ASC | Tennis balls vs. Bottles | | NA | NA | NA | NA |
|  | Tennis balls vs. Bricks | | NA | NA | NA | NA |
|  | Bottles vs. Bricks | | 26.150 | -112.885 | 165.185 | 1.000 |
| SCO | Tennis balls vs. Bottles | | -750.000 | -1082.357 | -417.643 | <0.001 |
|  | Tennis balls vs. Bricks | | NA | NA | NA | NA |
|  | Bottles vs. Bricks | | NA | NA | NA | NA |
| **Puzzle types presented second** | | | | | | |
| ASC | Tennis balls vs. Bottles | | 13.323 | -110.349 | 136.995 | 1.000 |
|  | Tennis balls vs. Bricks | | 35.400 | -222.043 | 292.843 | 1.000 |
|  | Bottles vs. Bricks | | 22.077 | -221.807 | 265.061 | 1.000 |
| SCO | Tennis balls vs. Bottles | | NA | NA | NA | NA |
|  | Tennis balls vs. Bricks | | NA | NA | NA | NA |
|  | Bottles vs. Bricks | | -47.500 | -335.330 | 240.330 | 1.000 |
| **Puzzle types presented third** | | | | | | |
| ASC | Tennis balls vs. Bottles | | 26.431 | -72.421 | 125.282 | 1.000 |
|  | Tennis balls vs. Bricks | | 134.897 | -15.631 | 285.426 | 0.128 |
|  | Bottles vs. Bricks | | 108.467 | -46.237 | 263.171 | 0.491 |
| SCO | Tennis balls vs. Bottles | | NA | NA | NA | NA |
|  | Tennis balls vs. Bricks | | NA | NA | NA | NA |
|  | Bottles vs. Bricks | | NA | NA | NA | NA |
| **Puzzle order for tennis balls** | | | | | | |
| ASC | First vs. Second | | NA | NA | NA | NA |
|  | First vs. Third | | NA | NA | NA | NA |
|  | Second vs. Third | | -11.831 | -135.502 | 111.841 | 1.000 |
| SCO | First vs. Second | | NA | NA | NA | NA |
|  | First vs. Third | | -637.000 | -969.357 | -304.643 | 0.006e-4 |
|  | Second vs. Third | | NA | NA | NA | NA |
| **Puzzle order for bottles** | | | | | | |
| ASC | First vs. Second | | -12.327 | -146.700 | 122.046 | 1.000 |
|  | First vs. Third | | -11.050 | -150.085 | 127.985 | 1.000 |
|  | Second vs. Third | | 1.277 | -97.574 | 100.128 | 1.000 |
| SCO | First vs. Second | | -17.500 | -305.330 | 270.330 | 1.000 |
|  | First vs. Third | | NA | NA | NA | NA |
|  | Second vs. Third | | NA | NA | NA | NA |
| **Puzzle order for bricks** | | | | | | |
|  | | | **Diff** | **Lwr** | **Upr** | **P adj** |
| ASC | First vs. Second | | -16.400 | -262.883 | 230.083 | 1.000 |
|  | First vs. Third | | 71.267 | -83.437 | 225.971 | 0.960 |
|  | Second vs. Third | | 87.667 | -183.702 | 359.035 | 0.999 |
| SCO | First vs. Second | | NA | NA | NA | NA |
|  | First vs. Third | | NA | NA | NA | NA |
|  | Second vs. Third | | NA | NA | NA | NA |
